# Mutation spectrum in South American Lynch syndrome families

**DOI:** 10.1186/1897-4287-11-18

**Published:** 2013-12-18

**Authors:** Mev Dominguez-Valentin, Mef Nilbert, Patrik Wernhoff, Francisco López-Köstner, Carlos Vaccaro, Carlos Sarroca, Edenir Ines Palmero, Alejandro Giraldo, Patricia Ashton-Prolla, Karin Alvarez, Alejandra Ferro, Florencia Neffa, Junea Caris, Dirce M Carraro, Benedito M Rossi

**Affiliations:** 1 The Danish HNPCC Register, Clinical Research Centre, Hvidovre Hospital, Copenhagen University, Hvidovre, Denmark; 2 Department of Oncology, Institute of Clinical Sciences, Lund University, Lund, Sweden; 3 Department of Experimental Medical Science, Unit of Muscle Biology, Lund Transgenic Core Facility/Reproductive Immunology, Lund University, Lund, Sweden; 4 Molecular Laboratory, Clinica Los Condes, Santiago, Chile; 5 Hereditary Cancer Program, Hospital Italiano, Buenos Aires, Argentina; 6 Hospital Fuerzas Armadas, Grupo Colaborativo Uruguay de Investigación de Afecciones Oncológicas Hereditarias (GCU), Montevideo, Uruguay; 7 Department of Oncogenetics, Barretos Cancer Hospital, Barretos, Brazil; 8 Facultad de Medicina de la Universidad del Sinú, Montería, Colombia; 9 Department of Genetics UFRGS, Hospital de Clínicas, Porto Alegre, Brazil; 10 Department of Molecular Oncogenetics, Laboratory of Genomics and Molecular Biology, AC Camargo Hospital, Sao Paulo, Brazil; 11 Hospital Sirio Libanes, Sao Paulo, Brazil

**Keywords:** Lynch syndrome, *MLH1*, *MSH2*, South America, Mutation

## Abstract

**Background:**

Genetic counselling and testing for Lynch syndrome have recently been introduced in several South American countries, though yet not available in the public health care system.

**Methods:**

We compiled data from publications and hereditary cancer registries to characterize the Lynch syndrome mutation spectrum in South America. In total, data from 267 families that fulfilled the Amsterdam criteria and/or the Bethesda guidelines from Argentina, Brazil, Chile, Colombia and Uruguay were included.

**Results:**

Disease-predisposing mutations were identified in 37% of the families and affected *MLH1* in 60% and *MSH2* in 40%. Half of the mutations have not previously been reported and potential founder effects were identified in Brazil and in Colombia.

**Conclusion:**

The South American Lynch syndrome mutation spectrum includes multiple new mutations, identifies potential founder effects and is useful for future development of genetic testing in this continent.

## Background

Since the initial reports on disease-predisposing mutations in the mismatch-repair (MMR) genes *MLH1* [MIM:120436], *MSH2* [MIM:609309] and *MSH6* [MIM:600678] in the early 1990’ies, a large number of studies have contributed to the establishment of the molecular map of Lynch syndrome with over 3,072 unique genetic MMR gene variants identified. These data are predominantly based on studies from North America, Europe and Asia. The mutations affect *MLH1* in 42%*, MSH2* in 33%, *MSH6* in 18% and *PMS2* in 8% [1]. Nonsense mutations, frameshift mutations and missense mutations predominate, whereas large genomic rearrangements and splice-site variants constitute <10% of the alterations [1].

The South American population is ethnically mixed from American Indian and European ancestors. In Uruguay and Argentina, European ancestry predominates. In Brazil, significant African and American Indians roots apply. In Chile, Colombia, Peru and Bolivia, Spanish colonist and American Indian ancestry influence the populations [2,3]. Mutation screening in South American families suspected of Lynch syndrome has identified disease-predisposing germline mutations in *MLH1* and *MSH2* in 16-45% of families that fulfill the Amsterdam criteria and/or the Bethesda guidelines [2-7]. Hereditary colorectal cancer registries have been established in Argentina, Brazil, Uruguay and Chile with the aim to collect and share data on the MMR gene mutation spectrum, identify potential founder mutations, interpret the role of unclassified genetic variants and to study cancer risks in the South American Lynch syndrome population. We used published data and unpublished register data to describe the mutation spectrum in South American Lynch syndrome families.

## Methods

### Ethics statement

All patients provided an informed consent for inclusion into the South American registers during genetic counseling sessions and is in compliance with the Helsinki Declaration.

### Patient selection

**Figure 1:**
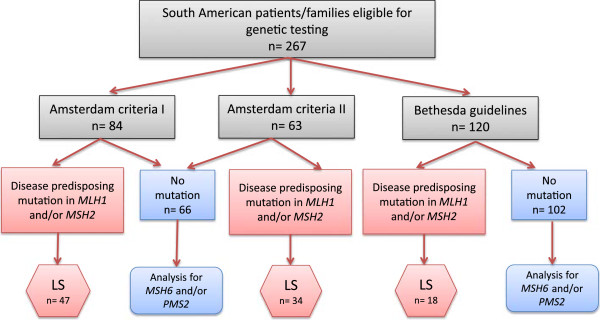
Flowchart of South American patients/families included in the study.

Families that fulfilled the Amsterdam criteria [8,9] and/or the Bethesda guidelines [10] were selected from the hereditary cancer registries at the Hospital Italiano (Buenos Aires, Argentina), the Hospital de las Fuerzas Armadas (Montevideo, Uruguay), the Clinica Los Condes (Santiago, Chile), the Barretos Cancer Hospital (Barretos, Brazil) and from two databases in Colombia and in Southeastern Brazil (Figure 1, Table 1) [2,3,5,11]. Patients were informed about their inclusion into the registries, which generally contained data on family history, age at onset and results of genetic testing.

**Table 1:** Summary of register data from MMR South American Lynch syndrome families

**South American Institutions**	**Total number of patients/families**	**MMR mutation carriers**	**% of MMR mutation carriers**	**Mean age at CRC diagnosis**	**Mean age at endometrial cancer diagnosis**
Hospital Italiano (Buenos Aires, Argentina)	28	14	50.0	44.3 (SD 6.2)	46.3 (SD 5.5)
Hospital de las Fuerzas Armadas (Montevideo, Uruguay)	25	7	28.0	35.1 (SD 7.6)	41.5 (SD 8.3)
Clinica Las Condes^a^ (Santiago, Chile)	50	20	40.0	35.7 (SD 10.7)	41.1 (SD 8.8)
Barretos Cancer Hospital^a^ (Barretos, Brazil)	23	15	65.2	39.4 (SD 13.8)	49.8 (SD 5.3)
Colombia^c^	13	8	61.5	NA	NA
Southeastern Brazil^b^	128	35	27.3	42.3 (SD 11.4)	48.8 (SD 2.4)
*Total*	267	99	37.1		

^a^MLPA analysis included, ^b^Valentin et al. 2011 [3] and Rossi et al. 2002 [5], ^c^Giraldo et al. 2005 [2] and Alonso-Espinaco et al. 2011 [11], *NA* Information not available, *MMR* mismatch-repair genes, *SD* standard deviation, *CRC* colorectal cancer.

### Disease-predisposing mutations

Methods to assess MMR status, e.g. microsatellite instability analysis and MMR protein staining, varied between the countries and were excluded from the present study since these data were incomplete. Molecular diagnosis was generally based on direct sequencing of *MLH1* and *MSH2*. Chilean and Brazilian families were also analyzed for large genomic rearrangements using the multiplex ligation-dependent probe amplification (MLPA) method (performed using the SALSA kit P003, MRC-Holland, Amsterdam, Netherlands).

### Mutation nomenclature

Mutation nomenclature was in accordance with the Human Genome Variation Society (HGVS) guidelines [12]. Mutations in the *MLH1* or *MSH2* genes were considered deleterious if they: a) were classified as pathogenic in LOVD database; b) introduced a premature stop codon in the protein sequence (nonsense or frameshift mutation); c) occurred at donor or acceptor splice sites; or d) represented whole-exon deletions or duplications. All identified mutations were correlated to the MMR Gene Unclassified Variants Database (http://www.mmruv.info), the Mismatch Repair Genes Variant Database (http://www.med.mun.ca/mmrvariants/), the French MMR network (http://www.umd.be/MMR.html) and the International Society for Gastrointestinal Hereditary Tumors (InSIGHT) (http://www.insight-group.org).

### Variants of uncertain significance

To establish the pathogenicity of variants of uncertain significance, web-based programs, i.e. Polyphen, MAPP-MMR, SIFT, P-mut and PON-MMR were applied to predict the effect of an amino acid substitution based on protein structural change and/or evolutionary conservation [13-17].

### Statistical analysis

The statistical analyses were performed using the statistical software package IBM SPSS Statistics 20 (SPSS, Chicago, IL, USA).

## Results

In total, 110 families harbored MMR gene variants, of which 99 were classified as Lynch syndrome predisposing and 11 were regarded as variants of uncertain significance. Mutations in *MLH1* and *MSH2* were identified in 37% (range 27-65% in the different countries/registries) of the families that fulfilled the Amsterdam criteria and/or Bethesda guidelines (Table 1). When the Amsterdam criteria were considered, the mutation detection rate was 55% (81/147), whereas 15% families that fulfilled the Bethesda guidelines had disease-predisposing mutations. The mean age at diagnosis was 35–44 years for colorectal cancer and 41–49 years for endometrial cancer in the different registries (Table 1). Pedigree information was available from 54 families and showed that among the Lynch syndrome-associated tumors, 65% were colorectal cancers (of which 43% were located in the right side of the colon), 22% endometrial cancers and 13% constituted other Lynch syndrome-associated cancer types.

**Figure 2:**
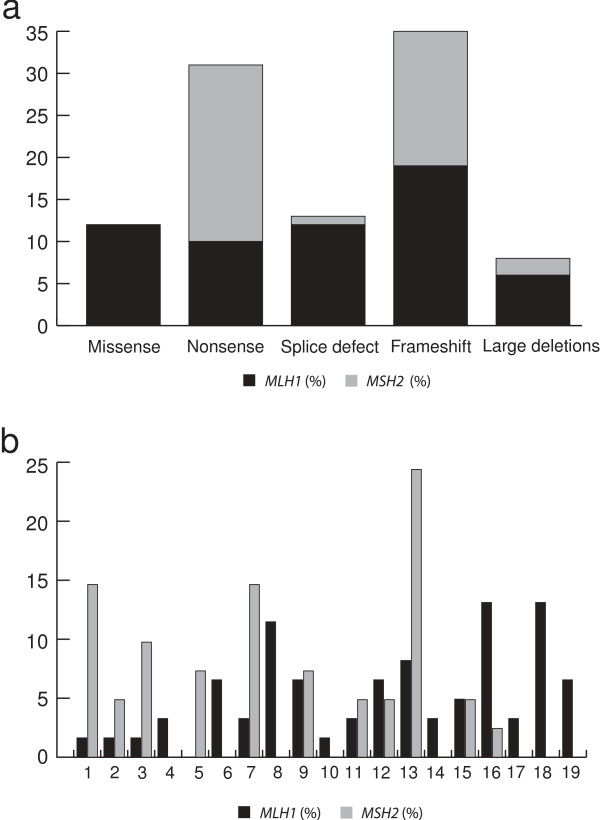
**Spectrum of pathogenic mutations in *MLH1 *** **and *MSH2 *** **genes a) Types of pathogenic germline mutations; b) Distribution along all exons of the *MMR *** **genes.**

Of the 99 disease-predisposing MMR gene mutations, 60% affected *MLH1* and 40% affected *MSH2* (Table 2). Frameshift and nonsense mutations were the most common alterations (36% and 31%, respectively), followed by splice site mutations (13%), missense mutations (12%) and large deletions (8%) (Figure 2a)*.* Though the mutations were spread over the genes, hot-spot regions included exons 16 and 18 in *MLH1* (13% of the mutations each) and exon 13 in *MSH2* (24% of the mutations each) (Figure 2b).

**Table 2:** Spectrum of alterations in South American Lynch syndrome families

**Gene**	**Nucleotide**	**Consequence**	**Exon**	**Reported as**	**Country**	**Number of families**	**References**
*MLH1*	c.1-?_116 + ?del	p.M1_C39 > FfsX13	1	Causal	Chile	2	InSIGHT
	c.199G > A	p.G67R	2	Causal	Argentina	1	InSIGHT
	c.211G > T	p.E71X	3	Causal	Brazil	1	InSIGHT
	c.289 T > G	p.Y97D	3	VUS	Uruguay	1	InSIGHT
	C.336 T > A	p.H112Q	4	VUS	Argentina	1	InSIGHT
	c.350C > T	p.T117M	4	Causal	Uruguay	2	InSIGHT
	c.421C > G	p.P141A	5	VUS	Colombia	1	Giraldo et al. 2005 [2]
	c.503dupA	p.N168KfsX4	6	Causal	Chile	1	InSIGHT
	c.503delA^a^	p.N168IfsX34	6	Causal	Brazil	1	Not previously described
	c.545 + 3A > G		6	Causal	Brazil	2	InSIGHT
	c.588 + 2 T > A^a^		7	Causal	Brazil	1	Valentin et al. 2011 [3]
	c.588 + 5G > C		7	Causal	Brazil	1	InSIGHT
	c.665delA	p.N222MfsX7	8	Causal	Uruguay	2	InSIGHT
	c.676C > T	p.R226X	8	Causal	Argentina	1	InSIGHT
	c.677G > A	p.R226Q	8	Causal	Argentina, Brazil	3	InSIGHT
	c.677 + 5G > A		8	Likely causal	Chile	1	French MMR network
	c.779 T > G	p.L260R	9	Causal	Brazil	1	InSIGHT
	c.790 + 1G > A		9	Causal	Chile, Colombia	3	InSIGHT
	c.791-6_793delgtttagATC^a^		10	Causal	Brazil	1	Valentin et al. 2011 [3]
	c.794G > C	p.R265P	10	VUS	Chile	1	InSIGHT
	c.901C > T	p.Q301X	11	Causal	Chile	1	InSIGHT
	c.1013A > G^a^	p.N338S	11	VUS	Brazil	1	InSIGHT
	c.1038 + 1G > T^a^	p.Y347FfsX13	11	Causal	Chile	1	Wielandt et al. 2012
	c.1039-8T_1558?896Tdup^a^	p.520Vfs564X	12 to 13	Causal	Colombia	2	Alonso-Espinaco et al. 2011 [11]
	c.1276C > T	p.Q426X	12	Causal	Brazil	3	InSIGHT
	c.1459C > T	p.R487X	13	Causal	Brazil	1	InSIGHT
	c.1499_1501delTCA^a^	p.I500del	13	Causal	Brazil	1	Rossi et al. 2002 [5]
	c.1558 + 1G > T		13	Causal	Brazil	1	InSIGHT
	c.1558 + 14G > A		13	VUS	Colombia	2	InSIGHT
	c.1559-2A > C		13	Causal	Chile	1	InSIGHT
	c.1559-?_1731 + ?del	p.V520_S577 > GfsX7^b^	14 -15	Causal	Chile	1	Wielandt et al. 2012
	c.1639_1643dup TTATA^a^	p.L549YfsX44	14	Causal	Brazil	1	Valentin et al. 2011 [3]
	c.1690_1693delCTCA	p.L564FfsX26	15	Causal	Brazil	1	InSIGHT
	c.1724G > A	p.R575K	15	VUS	Argentina	1	InSIGHT
	c.1731 + 3A > T^a^	Skipping exon 15	15	Causal	Chile	1	Alvarez et al. 2010 [6]
	c.1846delAAG	p.K616del	16	Causal	Argentina	1	InSIGHT
	c.1852_1853delinsGC	p.K618A	16	Causal	Argentina	1	InSIGHT
	c.1852_1854 delAAG	p.K618del	16	Causal	Argentina	1	InSIGHT
	c.1853A > C	p.K618T	16	VUS	Brazil	1	InSIGHT
	c.1853delAinsTTCTT^a^	p.K618IfsX4	16	Causal	Brazil	2	Valentin et al. 2011 [3]
	c.1856delG^a^		16	Causal	Colombia	2	Giraldo et al. 2005 [2]
	c.1890dup^a^	p.D631fsX1	16	Causal	Argentina	1	Valentin et al. 2011 [3]
	c.1897-?_1989 + ?del^a^		17-19	Causal	Brazil	1	Not previously described
	c.1918C > T	p.P640T	17	VUS	Colombia	1	InSIGHT
	c.1975C > T	p.R659X	17	Causal	Brazil	1	InSIGHT
	c.1998G > A	p.W666X	18	Causal	Brazil	1	Rossi et al. 2012 [5]
	c.2027 T > C	p.L676P	18	Causal	Brazil	1	InSIGHT
	c.2041G > A	p.A681T	18	Likely causal	Chile, Brazil, Colombia	4	French MMR network
	c.2092_2093delTC	p.S698RfsX5	18	Causal	Chile	1	Alvarez et al. 2010 [6]
	c.2224C > T^a^	p.Q742X	19	Causal	Brazil	1	Valentin et al. 2011 [3]
	c.2252_2253dupAA	p.V752KfsX26	19	VUS	Brazil	1	InSIGHT
	c.2104-?_2271 + ?del^b^	p.S702_X757del	19	Causal	Chile	2	Wielandt et al. 2012
*MSH2*	c.71delA^a^	p.Q24fs	1	Causal	Brazil	1	Not previously described
	c.166G > T^a^	p.E56X	1	Causal	Argentina	1	InSIGHT
	c.174dupC^a^		1	Causal	Brazil	1	Not previously described
	c.175dupC^a^	p.K59QfsX23	1	Causal	Brazil	1	Valentin et al. 2011 [3]
	c.181C > T^a^	p.Q61X	1	Causal	Uruguay	1	Sarroca et al. 2003
	c.187delG	p.V63fsX1	1	Causal	Brazil	1	InSIGHT
	c.289C > T	p.Q97X	2	Causal	Argentina	1	InSIGHT
	c.212-?_366 + ?del	p.A72_K122 > FfsX9	2	Causal	Chile	1	InSIGHT
	c.388_389delCA	p.Q130VfsX2	3	Causal	Brazil, Argentina	2	InSIGHT
	c.530_531delAA^a^	p.E177fsX3	3	Causal	Uruguay	1	Sarroca et al. 2003
	c.596delTG^a^		3	Causal	Colombia	1	Giraldo et al. 2005 [2]
	c.862C > T	p.Q287X	5	Causal	Brazil	1	InSIGHT
	c.897 T > G	p.Y299X	5	Causal	Chile	1	Wielandt et al. 2012
	c.942 + 3 A > T		5	Causal	Brazil	1	InSIGHT
	c.1077-?_1276 + ?del	p.L360KfsX16	7	Causal	Argentina	1	InSIGHT
	c.1147C > T	p.R382X	7	Causal	Brazil	1	InSIGHT
	c.1215C > A	p.Y405X	7	Causal	Chile	1	InSIGHT
	c.1216C > T	p.R406X	7	Causal	Uruguay	1	InSIGHT
	c.1249delG	p.V417LfsX21	7	Causal	Brazil	1	InSIGHT
	c.1255C > T	p.Q419X	7	Causal	Brazil	1	InSIGHT
	c.1444A > T^a^	p.R482X	9	Causal	Brazil	1	Valentin et al. 2011 [3]
	c.1447G > T	p.E483X	9	Causal	Brazil	2	InSIGHT
	c.1667delT^a^	p.L556X	11	Causal	Brazil	1	Valentin et al. 2011 [3]
	c.1667_1668insA^a^	p.T557DfsX5	11	Causal	Brazil	1	Rossi et al. 2002 [5]
	c.1910delC^a^	p.R638GfsX47	12	Causal	Argentina	1	Vaccaro et al. 2007
	c.1967_1970dupACTT^a^	p.F657LfsX3	12	Causal	Brazil	1	Valentin et al. 2011 [3]
	c.2038C > T	p.R680X	13	Causal	Chile	1	InSIGHT
	c.2046_2047delTG^a^	p.V684Dfs*14	13	Causal	Argentina	1	InSIGHT
	c.2131C > T	p.R711X	13	Causal	Brazil	1	InSIGHT
	c.2152C > T	p.Q718X	13	Causal	Brazil	6	InSIGHT
	c.2185_2192del7insCCCT^a^	p.M729_E731delinsP729_X730	13	Causal	Chile	1	Alvarez et al. 2010 [6]
	c.2187G > T^a^	p.M729I	13	VUS	Brazil	1	Valentin et al. 2011 [3]
	c.2525_2526delAG^a^	p.E842VfsX3	15	Causal	Brazil	2	Valentin et al. 2011 [3]
	c.2785C > T^a^	p.R929X	16	Causal	Brazil	1	Valentin et al. 2011 [3]

^a^First reported, *VUS* variants of unclassified significance, MLH1 (MIM#120436), MSH2 (MIM#609309),^b^pathogenecity demonstration ongoing.

**Figure 3:**
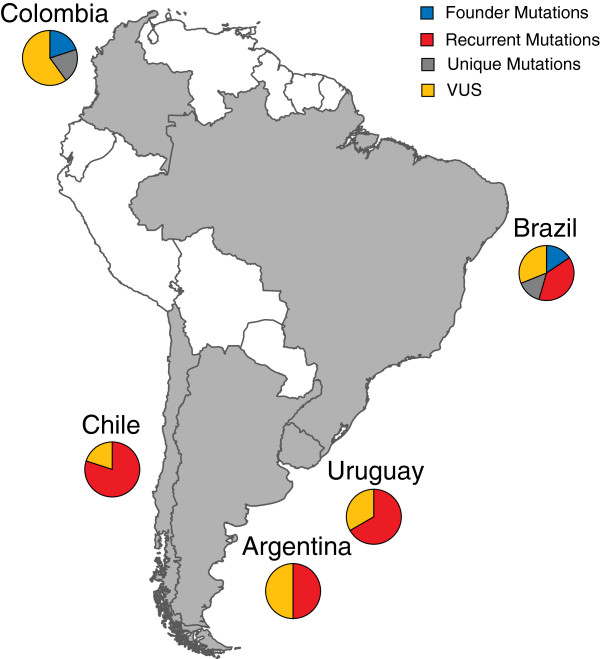
**Map of South America showing the countries where Lynch syndrome families with the founder, recurrent, unique mutations and variants of unclassified significance (VUS) have been identified.** The figure depicts the countries participating in the study (gray). The pie chart represents in percentage the recurrent mutations, unique mutations, founder mutations and VUS identified in the South American families. Brazil is characterized by 16% of the founder mutations, 39% of the recurrent mutations, 14% of the unique mutations and 31% of the VUS, while Colombia by 20% of the founder mutations, 20% of the unique mutations and 60% of the VUS. Chile, Argentina and Uruguay are characterized by 80%, 50% and 67% of the recurrent mutations and 20%, 50% and 33% of the VUS, respectively.

In total, 10 mutations identified in at least two South American families were classified as recurrent. Among these, the *MSH2* c.2152C > T identified in Brazil represents an internationally hot-spot. Three founder mutations were identified in five South American families. The *MLH1* c.545 + 3A > G and the *MSH2* c.942 + 3A > T have been identified as founder mutations in Italy and in Newfoundland and were also identified in Brazilian families [3]. The *MLH1* c.1039-8T_1558 + 896Tdup has been suggested to represent a founder mutation in Colombia [2,11]. Mutations that were unique and herein first reported in more than one family included the *MLH1* c.1853delAinsTTCTT in Brazil, the *MLH1* c.1856delG in Colombia and the *MSH2* c.25252_2526delAG in Brazil (Table 2) (Figure 3).

In total, 11 variants of unclassified significance were identified in individuals from Argentina, Uruguay, Chile, Brazil and Colombia (Table 3) [2,3]. *In silico* analysis suggested that the *MLH1* c.289 T > G, the *MLH1*c.794G > C and the *MLH1*c.1918C > T were likely disease-predisposing (Table 3).

**Table 3:** Variants of unclassified significance and in silico prediction in South American Lynch syndrome families

**Country**	**Gene**	**Nucleotide**	**Consequence**	**Exon**	**Polyphen**		**SIFT**		**MAP_MMR**		**P-mut**		**PON-MMR**	
					**Score**	**Classification**	**Score**	**Classification**	**Score**	**Classification**	**Score**	**Classification**	**Score**	**Classification**
Uruguay	*MLH1*	c.289 T > G	p.Y97D	3	0.999	Probably damaging	0	Damaging	10.51	Damaging	0.7266	Pathological	0.83	Pathogenic
Argentina	*MLH1*	c.336 T > A	p.H112Q	4	1	Probably damaging	0.03	Damaging	2.430	Neutral	NA	NA	0.61	VUS
Colombia	*MLH1*	c.421C > G	p.P141A	5	0.329	Benign	0.05	Damaging	3.15	Borderline deleterious	0.4928	Neutral	0.48	VUS
Chile	*MLH1*	c.794G > C	p.R265P	10	1	Probably damaging	0	Damaging	38.09	Damaging	0.7623	Pathological	0.83	Pathogenic
Brazil	*MLH1*	c.1013A > G	p.N338S	11	0.506	Possibly Damaging	0.05	Damaging	2.78	Neutral	0.2551	Neutral	0.38	VUS
Colombia	*MLH1*	c.1558 + 14G > A		13	NA	NA	NA	NA	NA	NA	NA	NA	NA	NA
Argentina	*MLH1*	c.1724G > A	p.R575K	15	0.001	Benign	0.40	Tolerated	1.490	Neutral	NA	NA	0.15	Neutral
Brazil	*MLH1*	c.1853A > C	p.K618T	16	0.997	Probably damaging	0.02	Damaging	5.11	Damaging	0.7802	Pathological	0.67	VUS
Colombia	*MLH1*	c.1918C > T	p.P640T	17	1	Probably damaging	0	Damaging	17.77	Damaging	0.6534	Pathological	0.83	Pathogenic
Brazil	*MLH1*	c.2252_2253dupAA	p.V752Kfs*26	19	NA	NA	NA	NA	NA	NA	NA	NA	NA	NA
Brazil	*MSH2*	c.2187G > T	p.M729I	13	2.293	Probably damaging	0	Damaging	21.99	Damaging	0.1988	Neutral	0.71	VUS

MLH1 (MIM#120436), MSH2 (MIM#609309), NA: not applicable, VUS: variants of unclassified significance, If SIFT score <0.05 then the aminoacid (AA) substitution is predicted to affect protein function, if PolyPhen score >0.5 then the AA substitution is predicted to affect protein function, if MAPP-MMR score >4.55 then the AA substitution is predicted to affect protein function, If P-mut score > 0.5, the AA substitution is classified as pathological, if PON-MMR score >0.7615, the AA substitution is classified as pathogenic.

## Discussion

In South America, disease-predisposing mutations linked to Lynch syndrome have been identified in 99 families, which corresponds to 37% of the families that fulfilled the Amsterdam criteria and/or Bethesda guidelines and underwent genetic testing. The mutation rate is high compared to prevalence rates of 28% for *MLH1* and 18% for *MSH2* in the Asian population, 31% and 20% in a multi-ethnic American population and 26% and 19% in European/Australian populations [18]. The mutation spectrum is predominated by *MLH1* (60%) and *MSH2* (40%) mutations [3,19-22], but the seemingly larger contribution than the 42% and 33% reported in the InSIGHT database could reflect failure to test for *MSH6* and *PMS2* mutations in most South American studies [1]. Referral bias in populations that have more recently been screened for mutations represents a potential limitation, but the strong contribution from *MLH1* and *MSH2* could also reflect population structure [2,4,5,7]. Frameshift mutations and nonsense mutations were the most common types of mutations, which are in agreement with findings from other populations [1,23-26], with hotspots in exons 16 and 18 of *MLH1* and in exon 13 of *MSH2* (Figure 2b). Exon 16 and 18 in *MLH1* has been identified as a genetic hot spot also in other populations with 26% of the *MLH1* mutations reported herein [3,18]. The frequent mutations in *MSH2* exon 13 may be linked to the c.2152C>, which was first identified in Portuguese Lynch syndrome families. This alteration accounted for 35% (6/17) of the *MSH2* mutations in the Brazilian population, which is in line with the Portuguese migration to Brazil [3,27].

Founder mutations have been identified in several populations where they significantly contribute to disease predisposition and thereby allow for directed genetic testing [28]. Two of the mutations identified in South American Lynch syndrome families have been suggested to constitute potential founder mutations in other populations, e.g. the Italian *MLH1* c.545 + 3A > G and the Newfoundland *MSH2* c.942 + 3A > T [3]. The Spanish founder mutations *MLH1* c.306 + 5G > A and c.1865 T > A and *MSH2* c.2635-3 T > C; c2635-5C > T; c.2063 T > G were, however, not observed in South American Lynch syndrome families [27-30]. In Colombia, the *MSH2* c.1039-8T_1558 + 896Tdup was suggested to represent a founder mutation [2,11]. The Colombian population has a mixed ancestry with a strong influence from Spanish colonists and thereby genetically differs from previously studied populations [2,6].

## Conclusions

In conclusion, disease-predisposing mutations in *MLH1* and *MSH2* have been identified in a relatively large proportion of the South American families suspected of Lynch syndrome that have been tested. Genetic hot-spot regions, internationally recognized founder mutations and potential South American founder mutation have been recognized, which is of relevance for genetic counseling and testing that are increasingly available in South America.

## Competing interests

The authors declare that they have no competing interests.

## Authors’ contributions

MDV, MN, BMR participated in the conception and design of the study. All authors participated in the acquisition of data, or analysis, interpretation of data and have been involved in drafting the manuscript. All authors read and approved the final manuscript.
